# Ovariectomy upregulated the expression of Peroxiredoxin 1 & 5 in osteoblasts of mice

**DOI:** 10.1038/srep35995

**Published:** 2016-10-27

**Authors:** Juan Du, Wei Feng, Jing Sun, Cuijie Kang, Norio Amizuka, Minqi Li

**Affiliations:** 1 Department of Bone Metabolism, School of Stomatology Shandong University, Shandong Provincial Key Laboratory of Oral Tissue Regeneration, Jinan, 250012, China; 2 The Key Laboratory of Plant Cell Engineering and Germplasm Innovation of Ministry of Education, School of Life Sciences, Shandong University, Jinan, China; 3 Department of Developmental Biology of Hard Tissue, Graduate School of Dental Medicine, Hokkaido University, Sapporo, Japan

## Abstract

Peroxiredoxin (PRX), a family of peroxidases, is associated with various biological processes such as the detoxification of oxidants and cell apoptosis. Besides, the anti-apoptosis effect of estrogen results partially from its anti-oxidant function. The purpose of this study was to investigate the expression of PRXs in ovariectomy (OVX) mice and the related anti-oxidative mechanism of estrogen. Eight-week-old mice were subjected to ovariectomy. MC3T3-E1 cells were pretreatment with 17b-estradiol and N-acetyl cysteine followed by oxidative injury induced with H_2_O_2_. Western blot and real time-PCR were applied to clarify the expressions of PRX1 and caspase-3, with both wild-type and PRX1 knockout MC3T3-E1 cells generated by CRISPR/Cas9 technology. The results showed PRX1 and PRX5 were upregulated in osteoblasts in the proximal tibial metaphysis of ovariectomy mice. Interestingly, PRX1 and PRX5 showed different distribution patterns, with PRX1 mainly accumulated in cell nuclei and PRX5 in the cytoplasm. Gene expression analysis showed significantly reduced expressions of PRX1 and caspase-3 in the pretreatment groups when compared with cells treated with H_2_O_2_ alone. Also, a decrease of caspase-3 expressions was observed in PRX1 knockout MC3T3-E1 cells with or without H_2_O_2_ in comparison to wild-type cells. These findings suggested that PRX may play important roles in estrogen-deficient osteoporosis. (200 words).

Over the last 60 years estrogen deficiency has been highlighted as a key factor of osteoporosis in both women and men^1^. Recent mechanistic studies have shown that aging and the associated increase in reactive oxygen species (ROS) – the radical forms of oxygen – may act as the chief culprits underpinning the disease mechanism^2^,^3^,^4^,^5^,^6^. Indeed, the stimulatory effects of gonadectomy on oxidative stress, osteoclastogenesis and osteoblast apoptosis, as well as the loss of bone mass were attenuated by treatment with antioxidants such as NAC or ascorbate, which were similar to estrogens and androgens^3^,^4^,^5^.

ROS, including the superoxide radical (·O_2_^−^), hydrogen peroxide (H_2_O_2_) and hydroxyl radical (·OH), are continuously produced in the mitochondria as by-products under normal physiological conditions. Yet, excessive accumulation of ROS in the body causes damage to cell components such as the cell membrane, cytoplasm, and ultimately to DNA^7^. Cells scavenge ROS by production of anti-oxidative enzymes, such as superoxide dismutase (SOD), catalase and glutathione peroxidase (GPX) thus protecting cellular components from damage due to oxidative stress^8^.

Peroxiredoxin (PRX) is a family of peroxidases with molecular weight of 20 to 30 kDa^9^,^10^. They are found in organisms from all kingdoms and abundantly expressed in the cellular cytoplasm^10^. Though their catalytic efficiency is less than that of catalase and GPX, they typically exhibit a higher affinity towards H_2_O_2_ than other anti-oxidative enzymes^11^. Mammalian cells express at least six isoforms of PRX (from 1 to 6), which are classified into three subgroups (typical 2Cys, atypical 2Cys and 1Cys) based on the number and position of Cys residues that participate in catalysis^11^,^12^. Members of the typical 2Cys subgroup, including PRX1 through PRX4, contain an additional conserved cysteine in the carboxyl-terminal region, whereas PRX5 and PRX6, which are members of the atypical 2Cys and 1Cys subgroups, respectively, lack this second conserved cysteine^12^. In addition to their roles as peroxidases, a body of evidence has begun to accumulate to suggest that individual members also serve divergent functions associated with various biological processes, such as the cell functions, apoptosis and gene expression^8^.

Despite these advances, it remains unclear how estrogen deficiency may contribute to osteoporosis and whether PRXs are involved in this disease process. In this study, we aimed to investigate the expression of PRX1 and PRX5 in estrogen deficient mice and any potential anti-oxidative role that they may exert (Fig. 1).

## Materials and Methods

### Animal Experimentation

All animal experiments were conducted according to the Guidelines for Animal Experimentation of Shandong University. The animal care and experimental protocol were approved by a committee of the Medical Ethics Committee for Experimental Animals, Shandong University School of Stomatology. Total 24 female Kunming mice, 8 weeks old, weighting 20–25 g, 12 for each groups, were obtained from the Laboratory Animal Centre of Shandong University (Jinan, China) and kept in plastic cages under standard laboratory conditions. All mice were fed with a standard rodent diet ad libitum. Mice were subjected to OVX or a sham operation, followed by pair feeding. Four weeks after surgery, the mice were anesthetized with an intraperitoneal injection of 10% chloral hydrate (400 mg/100 g body weight) and fixed with 4% paraformaldehyde in 0.1 M phosphate buffer (pH 7.4) by transcardial perfusion. After fixation, tibiae were removed and immersed in the same fixative for an additional 24 h. Following that, samples were decalcified using a 10% EDTA-2Na solution for 3 weeks at 4 °C. The specimens were subsequently dehydrated through an ascending ethanol series and then embedded in paraffin using standard procedures. Serial longitudinal 5 μm thick sections were prepared for histological analysis using a rotary microtome (LEICA SM 2010R, Wetzlar, Germany).

### Histological examination with Hematoxylin and eosin staining

To identify the morphology of the proximal tibial metaphysis, hematoxylin and eosin (HE) staining was performed in OVX and SHAM groups. The slides were placed in the xylene to deparaffinage. Hydrate the tissue section by passing through decreasing concentration of alcohol baths and water. Stain in hematoxylin for 5 minutes then washed in water for 5 minutes. Stain in 1% Eosin Y for 10 minutes and wash in water for 5 minutes. Dehydrate in increasing concentration of alcohols and clear in xylene. Mount in mounting media. Stained sections were observed and digital images were taken with a light microscope (Olympus BX-53, Tokyo, Japan).

### TRAP staining for osteoclasts

To evaluate the localization, number and morphology of osteoclasts in the murine tibial sections, tartrate-resistant acid phosphatase (TRAP) staining was performed as previously published^13^. In brief, dewaxed paraffin sections were submerged in a mixture of 3.0 mg naphthol AS-BI phosphate, 18 mg red violet LB salt, and 100 mM L(+) tartaric acid (0.36 g) diluted in 30 ml of 0.1 M sodium acetate buffer (pH 5.0) for 15 min at 37 °C. The sections were then counterstained with methyl green for assessment by light microscopy (Olympus BX53, Tokyo, Japan).

### Immunohistochemistry for ALP, PRX1 and PRX5

Paraffin sections of 5 μm thickness were prepared for alkaline phosphatase (ALP), PRX1 and PRX5 immune labeling. Briefly, following xylene treatment, dewaxed paraffin sections were pretreated with 0.3% hydrogen peroxide for 30 min, and then blocked with 1% bovine serum albumin (BSA; Serological Proteins Inc., Kankakee, IL, USA) in PBS (1% BSA-PBS) for 20 min to reduce non-specific staining. The sections were then incubated for 2 h at room temperature with: (1) rabbit antiserum against rat tissue nonspecific ALP, generated by Oda *et al.*^14^ at a dilution of 1:100; (2) rabbit anti-PRX1 antibody (Abcam Ltd., Shanghai, China) at a dilution of 1:100; (3) rabbit anti-PRX5 antibody (Abcam Ltd., Shanghai, China) at a dilution of 1:100 in 1% BSA-PBS. After rinsing with PBS, the sections were incubated with horseradish peroxidase-conjugated swine anti-rabbit IgG (DaKo, Glostrup, Denmark) for 1 h at room temperature. The immunoreaction was visualized with diaminobenzidine (DAB) (Sigma-Aldrich, St. Louis, MO, USA). All sections were counter stained with methyl green and observed under a light microscope (Olympus BX53, Tokyo, Japan).

### Cell Culture

MC3T3-E1 mouse pre-osteoblast cell line was bought from the Shanghai Cell Center. The passage numbers of cell lines used for the experiments were from 2 to 10. MC3T3-E1 cells were seeded at the density of 4 × 10^5^ cells/well in a six-well polystyrene plate. Cells were cultured in the phenol red-free alpha-minimum essential medium (a-MEM; Hyclone, Logan, UT, USA) with 10% fetal bovine serum (FBS; Gibco, Thermo Fisher Scientific Inc., Waltham, MA, USA) and 1% penicillin/streptomycin. Cultures were maintained at 37 °C in a fully humidified atmosphere of 5% CO_2_ in air. The culture medium was changed every 3 days. 17b-estradiol (E_2_; Sigma-Aldrich, St. Louis, MO, USA) was added at a concentration of 10^−7^ M. N-Acetyl Cysteine (NAC; MedChem Express, USA) was added at a concentration of 30mM. The concentration was chosen based on the manufacturer’s recommendation and previously published studies. Following initial cell seeding, cells were incubated for 24 h. Then cells were cultured in phenol red-free a-MEM without FBS for 12 h to eliminate the estrogenic action before being pretreated with vehicle or reagents for 1 h^15^,^16^. Cells were then treated with vehicle or 0.2 mM H_2_O_2_ 20 min for real time-PCR, or 30 min for cell immunofluorescence and Western blot analysis.

### Cell Immunofluorescence

Cells were grown, treated, fixed and stained directly on coverslips. Cells were washed with PBS once. Then cells were immediately fixed with ice cold methanol for 10 min. Block specimen in 1% BSA-PBST (PBS + 0.1% Tween 20) for 60 min. Apply diluted rabbit anti-PRX1 primary antibody (Abcam Ltd., Shanghai, China) at a dilution of 1:100 in 1% BSA-PBST. Incubate overnight at 4 °C. Incubate cells with the FITC- Goat Anti- Rabbit IgG secondary antibody (Proteintech, Wuhan, Hubei, China) at a dilution of 1:200 in 1% BSA-PBST for 1 h at room temperature in the dark. Decant the secondary antibody solution and wash three times with PBS for 5 min each in the dark. Incubate cells with Hoechst 33342 (Beyotime Institute of Biotechnology, China) for 5 min. Mount coverslip with a drop of glycerinum. Images were captured by fluorescence microscopy (Olympus BX53, Tokyo, Japan).

### Construction of MC3T3-E1 strains with a knockout PRX1 gene by the CRISPR/Cas9 system

In this study, MC3T3-E1 mouse pre-osteoblast cell line was employed as the host strain for the inactivation of the PRX1 gene using the CRISPR/Cas9 system. The gRNAs targeting PRX1 genes were designed by online tool developed by Prof. Feng Zhang (http://crispr.mit.edu/). Then the DNA sequences expressing gRNAs of PRX1 genes were cloned into pGK1.1 linear vector (cat.no. GP0134). And the DNA sequences are: H-Prdx1-seqF: GACCTCAACTTACAGCAACT; H-Prdx1-seqR: ATTTCACGTACCACATAGAC. Cells was transfected with pGK1.1-PRX1 by electroporation. 48 hours after transfection, the genomic DNA were extract using DNeasy Blood & Tissue Kit (QIAGEN, USA). Then, the Cruiser™ Enzyme (Genloci Biotechnologies Inc., Nanjing, China) was used to find the positive clone. All of the colonies were tested by colony PCR using primers H-Prdx1-seqF and H-Prdx1-seqR. The complete deletion the PRX1 gene was confirmed by TA cloning of the PCR products.

### RNA isolation and gene expression by real-time polymerase chain reaction analysis (real-time PCR)

Total cell RNA was isolated from cells cultured after 14days with TRIzol (Invitrogen, Carlsbad CA, USA) reagent. Then, cDNA was synthesized from 1μg of total RNA using PrimeScriptTM RT reagent kit (Takara Bio, Shiga, Japan) according to manufacturer’s instruction. Highly purified gene-specific primers for PRX1, caspase-3 and GAPDH, were synthesized commercially (Shengong, Co. Ltd. Shanghai, China). Real-time PCR was performed using 1 μl cDNA template in a 10 μl volume with Bio-Red MyiQ single color Real-time PCR system. The thermal profile for all reactions was as follows: 10 s at 9 °C, followed by 40 cycles of 5s at 95 °C, 31s at 58 °C and 30s at 72 °C. Data collection was enabled at 72 °C in each cycle. Each mRNA value was normalized to that of the house keeping gene, mouse GAPDH. Results are reported as relative gene expression. The fold-change in gene expression relative to the control was calculated by 2^−ΔΔCT^.

### Western Blot Analysis

MC3T3-E1 cells were harvested at selected time points and lysed in RIPA lysis buffer (ComWin Biotech Co., Ltd., Beijing, China). Protein samples (50 μg) were mixed with 1/4 volume of 5 × SDS loading buffer and boiled at 95 °C for 5 min. Following sodium dodecyl sulfate-polyacrylamide gel electrophoresis (SDS-PAGE), proteins were transferred to polyvinylidene difluoride (PVDF) membranes. Immunoblotting was performed with the following antibodies: rabbit anti-PRX1 antibody (Abcam Ltd., Shanghai, China) at a dilution of 1:1000, rabbit anti-caspase-3 antibody (Abcam Ltd., Shanghai, China) at a dilution of 1:2000 and mouse anti-GAPDH (Abcam Ltd., Shanghai, China) at a dilution of 1:2000, overnight at 4 °C. The secondary antibodies used were horseradish peroxidase-conjugated swine anti-rabbit IgG (DaKo, Glostrup, Denmark) for PRX1 and caspase-3, and horseradish peroxidase-conjugated rabbit anti-mouse IgG (Abcam Ltd., Hong Kong) for GAPDH, at room temperature for 1h. Western blot images were captured using a FluorChem E System (ProteinSimple, Santa Clara, CA, USA). The blots were cropped from different gels but all the gels had been run under the same experimental conditions.

### Statistical Analysis

Image Pro Plus 6.2 (IPP 6.2) software (Media Cybernetics, Silver Spring, MD, USA) was used for counting the Trabecular area (Tb. Ar), Total bone area (T. Ar) and Trabecular perimeter (Tb. Pm). The ratio of Trabecular area (% Tb. Ar) was calculated using the formula (Tb. Ar/T. Ar) × 100. Trabecular Separation (Tb. Sp, μm) was calculated using the formula (2000/1.199) × (T. Ar-Tb. Ar)/Tb. Pm. The mean optical density of images was analyzed manually using the same software. Positive reaction areas of PRX1 and PRX5 were manually selected via a color cube-based manner. At least 10 sections from each sample were analyzed. 6 samples of each group were tested. All values are presented as mean ± standard deviation (SD). All specimens were reviewed in a blinded fashion by two pathologists. The differences between the SHAM group and OVX group were assessed by a Student’s *t*-test. Statistically significant values were considered *p* < 0.05.

## Results

### Effect of Estrogen Deficiency on Histological alterations of tibial metaphysis

**Figure 2:**
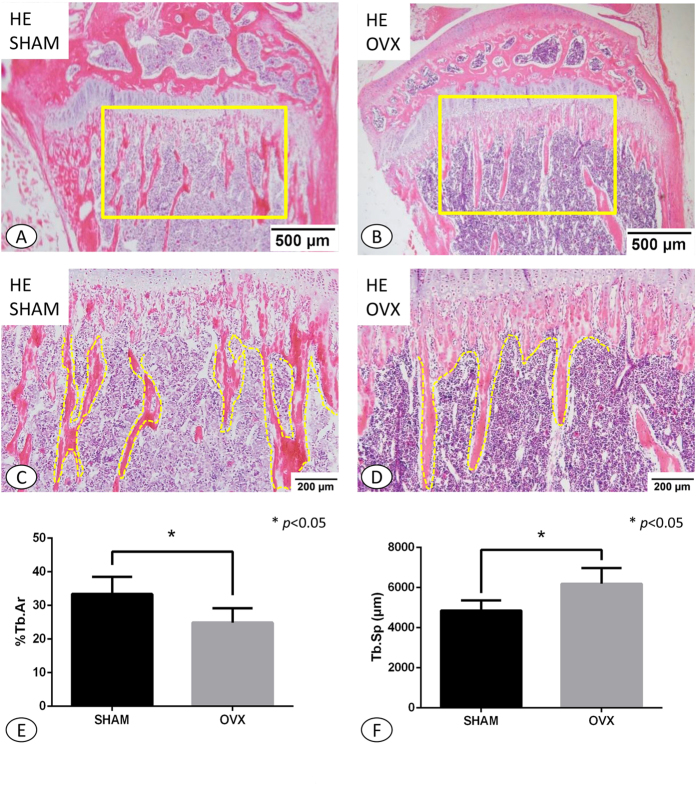
HE staining of proximal tibial metaphysis (**A**–**D**). **(A)** A lower magnification of proximal tibial metaphysis of the SHAM group. (**B)** A lower magnification of proximal tibial metaphysis of the OVX group. There was no significant difference of the width of cortical bone between the SHAM group and the OVX group. (**C**) Higher magnification of yellow frame in (**A**). The trabecular bone in SHAM group arranged irregularly and connected with each other to form a network. (**D)** Higher magnification of yellow frame in (**B**). The trabecular bone arranged regularly in the direction just identical to the mechanical loading direction in OVX group. (**E)** Statistical analysis of % Tb. Ar. (**F)** Statistical analysis of Tb. Sp. The % Tb. Ar was lower and the Tb. Sp was higher in OVX group compared with SHAM group. (**A**,**B**) ×40; (**C**,**D**) ×100. *p < 0.05.

HE staining showed no significant difference in the width of cortical bone between the OVX and SHAM groups (Fig. 2). However, the trabecular bone in the sham-operated control group appeared to be arranged irregularly forming a connective network of trabecular bone (Fig. 2A,C). While in the OVX group the trabecular bone showed a regular arrangement, with trabeculae only growing in the direction just identical to the mechanical loading direction (Fig. 2B,D). Statistical analysis revealed several significant differences between the OVX and SHAM groups, with the OVX group showing a decreased percentage trabecular area (% Tb. Ar: 33.39 ± 5.08% in SHAM group vs 24.91 ± 4.27% in the OVX group, *p* < 0.05, Fig. 2E) and increased trabecular separation (Tb. Sp: 4858.19 ± 508.28 μm in SHAM group vs 6188.23 ± 781.96 μm in the OVX group, *p* < 0.05, Fig. 2F).

### Effect of Estrogen Deficiency on Bone Remodeling Biomarkers of TRAP and ALP

**Figure 3:**
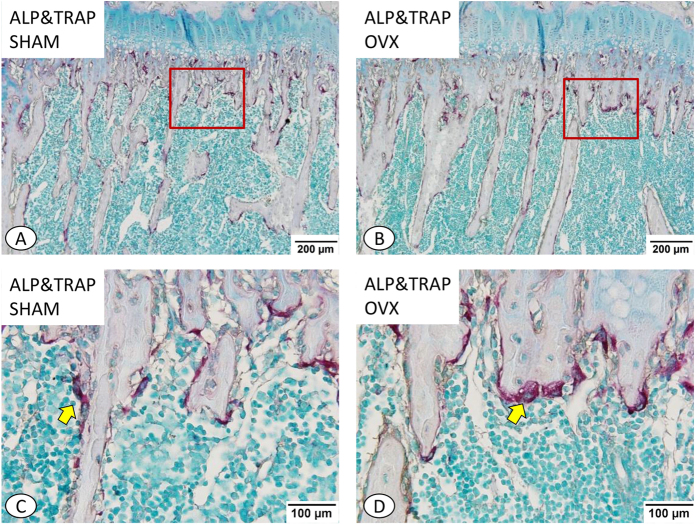
Double staining of TRAP and ALP of proximal tibial metaphysis (**A**–**D**). (**A**) A lower magnification of proximal tibial metaphysis of the SHAM group. (**B**) A lower magnification of proximal tibial metaphysis of the OVX group. The TRAP positive cells (red color) and ALP positive cells (brown color) were primarily observed on the surface of trabecular bone. The expression intensity of ALP seems a little higher in OVX group compared with the SHAM group. (**C**) Higher magnification of trabecular (red frame) in (**A**). (**D**) Higher magnification of trabecular (red frame) in (**B**). Many well-round TRAP positive cells (yellow arrow) were observed on the surface of trabecular bone in OVX group compared with that in SHAM group. In addition, it was found more apparently that the trabecular bone arranged irregularly in SHAM group and regularly in OVX group in these figures. (**A**,**B**) ×100; (**C**,**D**) ×200.

The double staining of ALP&TRAP showed that ALP positive cells were primarily observed on the surface of trabecular bone (Fig. 3). The expression intensity of ALP appeared to be slightly increased in the OVX group compared with the SHAM group (Fig. 3A,C). Images taken at high-magnification showed that an increased number of well-rounded TRAP positive cells were present on the surface of trabecular bone of the OVX group compared with that of the SHAM group (Fig. 3B,D). Furthermore, it was clear that the trabecular bone in SHAM group adopted the expected irregular arrangement irregularly, while the trabecular bone of OVX group showed a weaker regular structure.

### Effect of Estrogen Deficiency on PRX1 &PRX5 expressions

**Figure 4:**
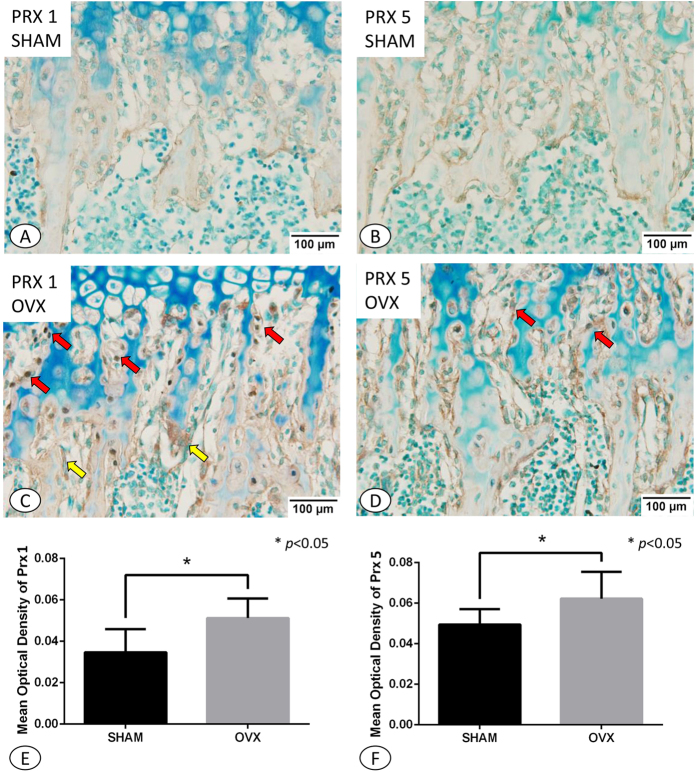
PRX1 (**A**,**C**) and PRX5 (**B**,**D**) immunohistochemistry in the trabecular bone. (**A)** PRX1 immunohistochemistry in trabecular region of the SHAM group. The positive expression of PRX1 was dominantly observed in the osteoblasts (red arrow) of metaphysis. PRX1 was located in the cytoplasm of the cells, but not in the nuclei. (**B)** PRX5 immunohistochemistry in trabecular region of the SHAM group. (**C)** PRX1 immunohistochemistry in trabecular region of the OVX group. PRX1 primarily located not only in the cytoplasm of osteoblasts (red arrow) but especially in their nuclei. The PRX1 positive expression was also found in osteoclasts (yellow arrow) (**D)** PRX5 immunohistochemistry in trabecular region of the OVX group. The expression of PRX5 mostly located in the cytoplasm of osteoblasts rather than their nuclei in both SHAM and OVX group. (**E)** Statistical analysis of mean optical density of PRX1. (**F)** Statistical analysis of mean optical density of PRX5. The expression intensities of both PRX1 and PRX5 in OVX group were much stronger than that in SHAM group. A-D, ×200. *p < 0.05.

Osteoblastic expression of both PRX1 and PRX5 was much stronger in samples from OVX group compared with samples from the SHAM group (Fig. 4). Interestingly, the positive expression of PRX1 was predominantly observed in the osteoblasts of the metaphysis (Fig. 4A,C). In mice from the SHAM group, PRX1 was located in the cellular cytoplasm and not in the nuclei (Fig. 4A). However, in comparison, samples from OVX mice showed PRX1 expression in both the cytoplasm and nuclei of osteoblasts. Osteoclasts from the OVX group also stained positive for PRX1 (Fig. 4C), yet this was not apparent within samples from the SHAM group.

PRX5 showed a similar expression pattern to that of PRX1, localized predominantly to the osteoblasts of the metaphysis, with stronger expression observed in the OVX group compared with the SHAM group. However, PRX5 was found to only localize to the cytoplasm of cells, with no nuclear expression observed in either the OVX or SHAM samples (Fig. 4B,D). Statistical analysis revealed significant differences in the mean optical density of PRX1 and PRX5 between the OVX and SHAM group (PRX1: 0.0347 ± 0.0112 in SHAM group vs 0.0512 ± 0.0095 in the OVX group, *p* < 0.05, Fig. 4E; PRX5: 0.0495 ± 0.0076 in SHAM group vs 0.0622 ± 0.0133 in the OVX group, *p* < 0.05, Fig. 4F).

### H_2_O_2_-induced apoptosis and PRX1 nuclei accumulation in MC3T3-E1 cells

**Figure 5:**
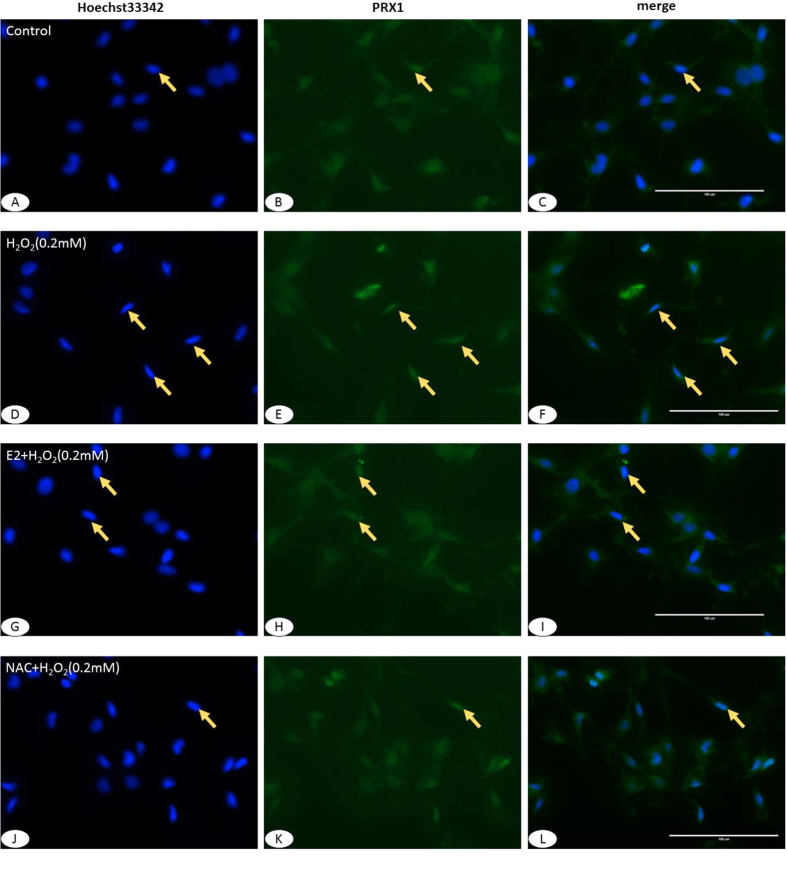
Cell Immunofluorescence of H_2_O_2_-induced expression of PRX1 and apoptosis in MC3T3-E1 cells. (**A–C)** MC3T3-E1 cell of Hoechst33342 staining (**A**), expression of PRX1 (**B**) and merge of (**A,B**) (**C**). (**D–F)** MC3T3-E1 cell after treated with H_2_O_2_ of Hoechst33342 staining (**D**), expression of PRX1 (**E**) and merge of (**D,E**) (**F**). (**G–I**) MC3T3-E1 cell after treated with E_2_ and H_2_O_2_ of Hoechst33342 staining (**G**), expression of PRX1 (**H**) and merge of (**G,H**) (**I**). (**J–L)** MC3T3-E1 cell after treated with NAC and H_2_O_2_ of Hoechst33342 staining (**J**), expression of PRX1 (**K**) and merge of (**J,K**) (**L**). After Hoechst33342 staining, a large number of cells in H_2_O_2_ group showed strong staining in the nuclei, whereas only a few cells in E_2_ group exhibited such changes. There were barely strong staining in the nuclei in control and NAC groups. Accordingly, the strong expression of PRX1 was accumulated in the nuclei of apoptotic-like cells. *Yellow arrow*: apoptosis-like cells. (**A**–**L**) ×400.

To evaluate the apoptosis of MC3T3-E1 cells after treated with H_2_O_2_ and the anti-apoptosis effect of E_2_, the Hoechst33342 staining was applied. A cell that is undergoing apoptosis demonstrates nuclear condensation and DNA fragmentation, which can be detected by staining with Hoechst 33342 and fluorescence microscopy^17^. And the Cell Immunofluorescence was used to verify the localization of the expression of PRX1 in cells. After Hoechst33342 staining, most of cells showed light staining in the nuclei with only occasional strong staining in control (Fig. 5A–C) and NAC groups (Fig. 5J–L). The expressions of PRX1 were mildly and evenly exhibited in the whole cells. However, a large number of cells in H_2_O_2_ group (Fig. 5D–F) showed strong staining in the nuclei and nuclear condensation, whereas only a few cells in E_2_ group (Fig. 5G–I) exhibited such changes. Accordingly, the strong expression of PRX1 was accumulated in the nuclei of apoptotic-like cells.

### 17b-Estradiol (E_2_) down-regulated the stimulated expressions of PRX1 and caspase-3 by H_2_O_2_

**Figure 6:**
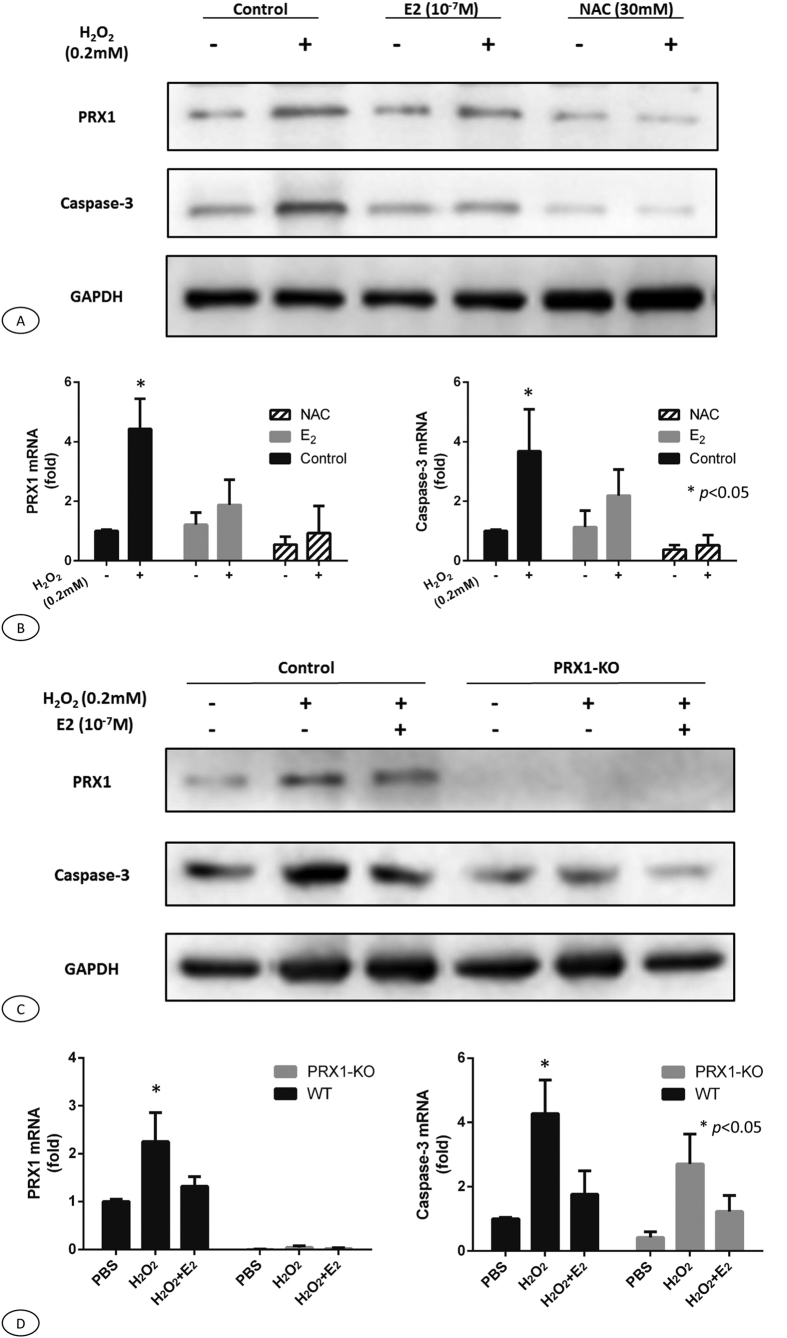
Western Blot and real time-PCR of H_2_O_2_-induced upregulation of PRX1 and caspase-3 expression in MC3T3-E1 cells. **(A)** Western blot analysis showed the pretreatment of osteoblast cells with E_2_ partially abrogate the increased PRX1 and caspase-3 expressions induced by H_2_O_2_. In addition, pretreatment of NAC could largely decreased the expression of caspase-3 and almost abolished the H_2_O_2_ induced upregulation of PRX1. (**B)** The mRNA expression of PRX1 and caspase-3. The results of RT-PCR were consistent with those of western blot. (**C)** Western blot analysis demonstrated that PRX1 proteins were not detectable in PRX1 knockout MC3T3-E1 osteoblast cells. Precondition with E_2_, attenuated the effect of H_2_O_2_ on caspase-3 expressions in both cells. (**D)** The mRNA expression of PRX1 and caspase-3. The results of RT-PCR were consistent with those of western blot. The blots were cropped from different gels but all the gels had been run under the same experimental conditions. *p < 0.05.

A basal level of PRX1 and caspase-3 expressions were initially noted in MC3T3-E1 murine pre-osteoblast cells. The exposure of MC3T3-E1 cells to H_2_O_2_ resulted in the upregulation of PRX1 and caspase-3 expressions at both the protein and mRNA levels (Fig. 6A,B). Treatment with E_2_ alone, had no observable effect on the expressions of PRX1 and caspase-3. However, pretreatment of these cells with E_2,_ appeared to partially abrogate the increased PRX1 and caspase-3 expressions induced by subsequent treatment with H_2_O_2_. In addition, pretreatment of NAC, a free radical scavenger, could largely decrease the expression of caspase-3 and almost abolish the H_2_O_2_ induced upregulation of PRX1 (Fig. 6A). The results of RT-PCR were consistent with those of western blot (Fig. 6B).

### PRX1 knockout decreased caspase-3 expressions of MC3T3-E1 cells

Western blot analysis demonstrated that PRX1 proteins were not detectable in PRX1 knockout MC3T3-E1 osteoblast cells (Fig. 6C). In addition, the caspase-3 level was lower than that of control group with and without precondition of H_2_O_2_. However, the pretreatment of these two kind of cells with E_2,_ appeared to attenuate the effect of H_2_O_2_ on caspase 3 expressions of MC3T3-E1 osteoblast cells. The results of RT-PCR were consistent with those of western blot (Fig. 6D).

## Discussion

In the present study, we employed ovariectomy mouse model to assess the effect of estrogen deficiency on bone and the expression of PRX. Moreover, PRX1 knockout cells generated by CRISPR/Cas9 technology were used to investigate the protective role of estrogen against H_2_O_2_ induced MC3T3-E1 osteoblast cell apoptosis. The results showed that PRX1 and PRX5 were up-regulated in the proximal tibial metaphysis of estrogen-deficient ovariectomy mice, which were especially observed in osteoblasts. Cell immunofluorescence demonstrated the upregulated expression of PRX1 was accumulated in the nuclei of Hoechst 33342 strong stained cells. Furthermore, preconditioning with E_2_ and NAC could attenuate upregulation of PRX1 and caspase-3 expression at both the mRNA and protein levels induced by H_2_O_2_. Also, a decrease of caspase-3 expression was observed in PRX1 knockout MC3T3-E1 cells with or without H_2_O_2_ in comparison to wild-type cells. These findings suggested that PRX may play important roles in estrogen-deficient osteoporosis.

Estrogen deficiency is associated with an increase in bone remodeling; increased osteoclastogenesis and osteoblastogenesis, increased osteoclast and osteoblast numbers, and correspondingly increased resorption and formation. When the amount of bone resorbed by osteoclasts is not fully restored by bone deposited by osteoblasts, this imbalance leads to a net loss in bone mass and strength^2^,^18^. In the present experiments, OVX mice showed an increased expression of ALP in osteoblasts accompanied by increased number of well-round TRAP positive cells compared with that in SHAM group, indicating a relatively higher bone turnover rate^5^,^19^. These led to a significantly increased level of trabecular separation and a significantly decreased percentage of trabecular area in OVX group compared with sham-operated control animals. Interestingly, the trabecular connectivity observed in estrogen deficient mice, as a result of OVX, was remarkably changed. The trabecular bone in the SHAM group arranged irregularly and showed good connectivity to form a network, while in the OVX group the trabecular bone was arranged regularly according to the direction of mechanical loading. Ding *et al.*^20^ applied micro-CT analysis to determine a significant increase in the intensity of the trabecular orientation in the primary trabecular direction and a strong trend of decreasing trabecular connectivity with age. The subtle reduction in the transverse direction increases the intensity of the trabecular in the loading axis, so this structural change could be effective in resisting loading^21^.

Estrogen deficiency impairs antioxidant system and promotes an increase of oxidative stress in bone, leading to disturbance of bone remolding and finally to osteoporosis^3^,^22^,^23^. It has been reported that the level of H_2_O_2_ increased while antioxidant levels, such as SOD, glutathione (GSH) and glutathione reductase (GSR), decreased in rodents after OVX. In another side, the upregulation of some PRXs in cells and tissues by various stress agents is considered an important biological response to prevent oxidative damages^24^. However, mammalian cells express at least six isoforms of PRX (from 1 to 6). And they exhibit different expression patterns during development; localizing dissimilarly within cells, and yielding different reaction intermediates during catalysis^10^,^25^. Interestingly, our results indicate that the expressions of PRX1 and PRX5 increased and had different distribution patterns in osteoblast cell structures in estrogen deficient mice, with PRX1 mainly accumulating in cell nuclei while PRX5 resides in the cytoplasm. The high levels of PRX expression after OVX are in accordance with previously published research, in which PRX1 expression levels in breast cancer cell lines were demonstrated to be higher than in normal or pseudonormal breast cell lines, and nuclear PRX1 levels were higher than cytoplasmic levels in these cancer cells^26^. To our knowledge, this is the first report that illustrates the divergent localization of PRX1 and PRX5 in osteoblasts. These may imply that oxidative stress caused by estrogen deficiency enhanced the expression of PRXs which not just played a role as an anti-oxidant enzyme during this procedure. Furthermore, it is indicated that various members of the PRX family possess different cellular functions as part of the mechanism of estrogen-deficient osteoporosis.

The protective effect of estrogen on osteoblasts against apoptosis might be associated with attenuating the generation of ROS^5^,^16^,^27^. NAC, a free radical scavenger, has been demonstrated to protect osteoblasts against hypoxic-stress-induced apoptosis^28^,^29^. Moreover, administration of antioxidants like NAC, as well as estrogens, prevents the OVX induced loss of bone mass^3^,^4^,^5^,^23^. In addition, caspase-3 is activated in the apoptotic cell both by extrinsic (death ligand) and intrinsic (mitochondrial) pathways, in which caspase-3 plays a dominant role^30^. In the current study, gene expression analysis showed significantly downregulated expressions of PRX1 and caspase-3 with pre-treatment of E_2_ and NAC when compared with those cells treated with H_2_O_2_ alone. Also, a decrease of caspase-3 expression was observed in PRX1 knockout MC3T3-E1 cells with or without H_2_O_2_ in comparison to wild-type cells. Beside, cell immunofluorescence demonstrated the upregulated expression of PRX1 was accumulated in the nuclei of Hoechst 33342 strong stained cells, which have been abolished by administration with E_2_ and NAC. Thus, it is suggested that H_2_O_2_ could increase the expression of apoptotic factor caspase-3 by enhancing PRX1 in MC3T3-E1 cell. These results indicate that estrogen likely protect osteoblasts from ROS induced apoptosis though attenuating the upregulation of PRX1. This was in consistent with a recent study which employed PRX1 null mice and found that PRX1 was not an effective protector against O3-induced oxidative damages^31^.

PRX1, previously termed OSF3, has already been shown to be specifically expressed in mouse osteoblastic cells^32^. Moreover, some isoforms of PRX appear to participate in signaling by controlling H_2_O_2_ concentration^10^,^12^. However, until now, the function of PRXs in regulating signaling pathways or cell cycle progression within osteoblasts has not been analyzed. Hansen *et al.*^33^ demonstrated in HeLa cells expressing nuclear localizing PRX1 (NLS-PRX1), stimulation with H_2_O_2_ (0.1–0.5 mM) enhanced NF-kB activation 1.8–2.8-fold, measured by a luciferase reporter assay. In contrast, PRX5 appears to differ from the two Cys-containing enzymes within this family, which includes PRX1, in terms of its intracellular localization, structure, and reaction mechanism^34^. Previous immunoblot analysis revealed that PRX5 is intracellularly localized to the cytosol, mitochondria, and peroxisomes^35^ and has been shown to inhibit the p53 induced generation of ROS and apoptosis of HeLa cells^36^.

**Figure 7:**
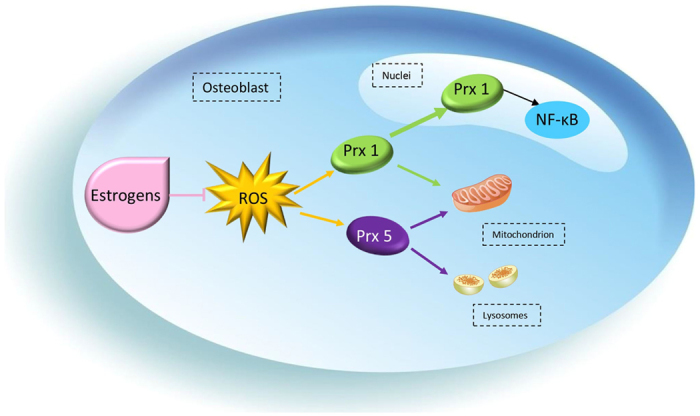
Schema of the relationship between Estrogen and ROS and the various roles of PRX1 & 5 in osteoblast.

The results of our experiments are summarized in a schema (Fig. 7). According to the evidence above, we propose that PRX1 may play an important role in the regulation of cell signaling pathways in the estrogen-deficient osteoporosis of mice, while PRX5 mainly serves as an oxidant scavenger. However, further research is required to interpret the specific functions of PRX1 & 5 in estrogen deficiency of mice.

We acknowledge that while these experiments may allow inferences on the roles of PRX enzymes in estrogen-deficient osteoporosis, we have not analyzed the function of these enzymes by osteoclast cells. So further work would be required to determine their specific function, particularly in the balance between the osteoblasts and osteoclasts. A further limitation of this work, is the time following OVX used in our experiments, for a more full analysis, multiple time points following the OVX operation would be examined to see if the observed effect is progressive in line with the development of osteoporosis.

## Conclusion

Expression of both PRX1 and PRX5 was increased in a murine estrogen-deficient model, implying that they may play a role in the development of estrogen-deficient osteoporosis. However, different members of the PRX enzyme family exert different roles in this pathological process, indicated by the differing expression patterns of each enzyme. While 17b-Estradiol (E_2_) had no effect alone on the expression of PRX1 in MC3T3-E1, it partially abrogated H_2_O_2_-induced upregulation of PRX1. We thus suggest that the association between estrogen and PRX1 may regulate osteoblast cell responses to oxidative stress.

## Additional Information

**How to cite this article**: Du, J. *et al.* Ovariectomy upregulated the expression of Peroxiredoxin 1 & 5 in osteoblasts of mice. *Sci. Rep.* **6**, 35995; doi: 10.1038/srep35995 (2016).

**Publisher's note**: Springer Nature remains neutral with regard to jurisdictional claims in published maps and institutional affiliations.

## Supplementary Material

Supplementary Information

## Figures and Tables

**Figure 1:**
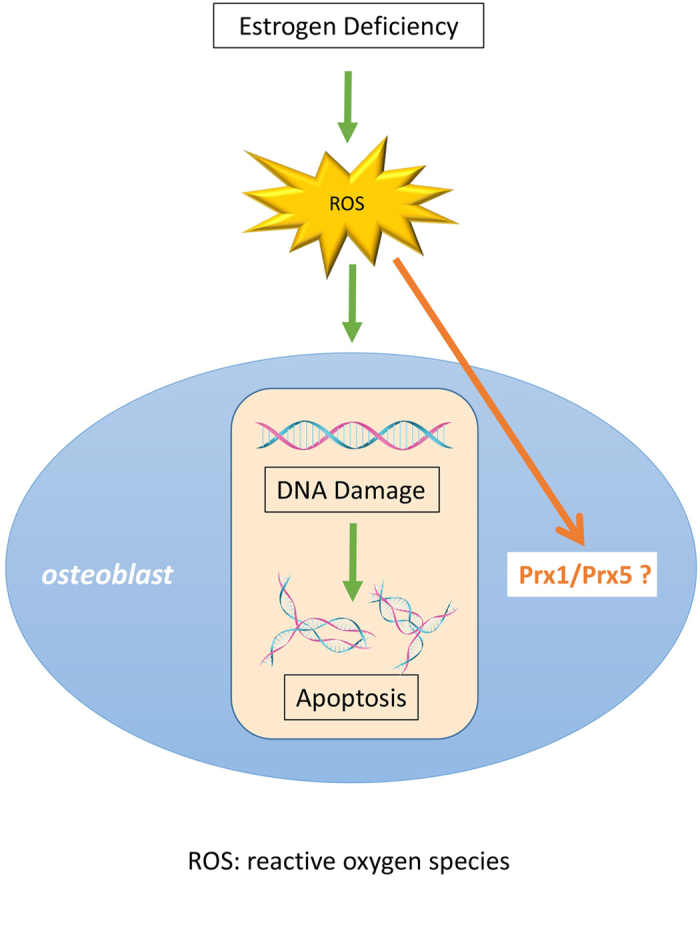
Schema of research background and purpose of this study. Excessive accumulation of ROS in the body, which causes by estrogen deficiency, damages cell DNA leading to cell death. Upregulation of some PRXs in cells is important biological response to cope with cell damages. The purpose of this study was to investigate the expression of PRX1 & PRX5 in mice after ovariectomy.
