# Supplementary material for: Ovariectomy upregulated the expression of Peroxiredoxin 1 & 5 in osteoblasts of mice
Source: Sci Rep. 2016 Oct 27;6:35995. doi: 10.1038/srep35995 (PMC5081527; doi:10.1038/srep35995)
Supplement: Supplementary Information [file srep35995-s1.pdf]

nature publishing group npg

In accordance to Nature Publishing Groups Authorship Policy we agree to change the authors of the manuscript as indicated below.

NAME OF JOURNAL: Scientific Reports

TITLE OF MANUSCRIPT: Ovariectomy upregulated the expression of Peroxiredoxin 1 & 5 in osteoblasts of mice

MANUSCRIPT NUMBER: SREP-16-19767A

CORRESPONDING AUTHORS NAME: Minqi Li

PREVIOUS AUTHOR NAMES:
Juan Du, Minqi Li

UPDATED AUTHOR NAMES:
Juan Du, Wei Feng, Jing Sun, Cuijie Kang, Norio Amizuka, Minqi Li

CHANGE TO AUTHOR LIST: Wei Feng, Jing Sun, Cuijie Kang and Norio Amizuka are newly added in our author list for their contributions to the modified manuscript.

Print Name	Signature	Date
Juan Du	Juan Du	2016. 9. 28
Wei Feng	Wei Feng	2016 9. 28
Jing Sun	Jing Sun	2016. 9.28
Cuijie Kang	Cuijie Kang	2016.9.28
Norio Amizuka	Norio Amizuka	2016. 9. 28
Minqi Li	Minqi Li	2016. 9. 28
